# Novel drug-resistance mechanisms of pemetrexed-treated non-small cell lung cancer

**DOI:** 10.18632/oncotarget.24704

**Published:** 2018-03-30

**Authors:** Ryosuke Tanino, Yukari Tsubata, Nanae Harashima, Mamoru Harada, Takeshi Isobe

**Affiliations:** ^1^ Division of Medical Oncology & Respiratory Medicine, Department of Internal Medicine, Faculty of Medicine, Shimane University, Shimane, Japan; ^2^ Laboratory of Biometabolic Chemistry, School of Health Sciences, Faculty of Medicine, University of the Ryukyus, Okinawa, Japan; ^3^ Department of Immunology, Faculty of Medicine, Shimane University, Shimane, Japan

**Keywords:** drug resistance, pemetrexed, NSCLC, EGFR-TKI, drug-induced senescence

## Abstract

Pemetrexed (PEM) improves the overall survival of patients with advanced non-small cell lung cancer (NSCLC) when administered as maintenance therapy. However, PEM resistance often appears during the therapy. Although thymidylate synthase is known to be responsible for PEM resistance, no other mechanisms have been investigated in detail. In this study, we explored new drug resistance mechanisms of PEM-treated NSCLC using two combinations of parental and PEM-resistant NSCLC cell lines from PC-9 and A549. PEM increased the apoptosis cells in parental PC-9 and the senescent cells in parental A549. However, such changes were not observed in the respective PEM-resistant cell lines. Quantitative RT-PCR analysis revealed that, besides an increased gene expression of thymidylate synthase in PEM-resistant PC-9 cells, the *solute carrier family 19 member1* (*SLC19A1)* gene expression was markedly decreased in PEM-resistant A549 cells. The siRNA-mediated knockdown of SLC19A1 endowed the parental cell lines with PEM resistance. Conversely, PEM-resistant PC-9 cells carrying an *epidermal growth factor receptor (EGFR)* mutation acquired resistance to a tyrosine kinase inhibitor erlotinib. Although erlotinib can inhibit the phosphorylation of EGFR and Erk, it is unable to suppress the phosphorylation of Akt in PEM-resistant PC-9 cells. Additionally, PEM-resistant PC-9 cells were less sensitive to the PI3K inhibitor LY294002 than parental PC-9 cells. These results indicate that SLC19A1 negatively regulates PEM resistance in NSCLC, and that EGFR-tyrosine-kinase-inhibitor resistance was acquired with PEM resistance through Akt activation in NSCLC harboring EGFR mutations.

## INTRODUCTION

Non-small cell lung cancer (NSCLC) is the most common type of lung cancer, and approximately two-thirds of patients with NSCLC are first encountered at the advanced stage. Usually, advanced-stage NSCLC patients without oncogenic driver mutations, such as a mutation of *epidermal growth factor receptor (EGFR)*, are treated primarily with platinum-based combination chemotherapy. As an alternative, pemetrexed (PEM) is an antifolate drug [1] that exerts anti-cancer effects on non-squamous NSCLC [2]. PEM has therapeutic advantages over gemcitabine when used with platinum-based chemotherapy to treat non-squamous NSCLC [3]. Platinum-based chemotherapy is repeated in four cycles, followed by the continuous administration of PEM alone. This continuous maintenance therapy has been conducted to prevent recurrence after platinum-based chemotherapy and improves the overall survival of advanced-stage non-squamous NSCLC patients [4]. However, such long-term therapy frequently results in the emergence of drug-resistant cancer cells, leading to cancer progression.

PEM targets several intracellular molecules; it inhibits cell proliferation by blocking synthesis of dTMP, and the main target is thymidylate synthase (TYMS) [5–10]. PEM has more effective to malignant pleural mesothelioma that has lower TYMS than high [11]. TYMS-overexpressing NSCLC cell lines show PEM resistance compared with parental cell lines [8]. However, a clinical report suggests no significant correlation between TYMS expression and PEM resistance of NSCLC [12]. Additionally, another report indicates no significant association between the TYMS expression and the clinicopathological factors of patients who received PEM as third- or fourth-line chemotherapy [13].

Solute carrier family 19 (folate transporter), member 1 (SLC19A1/RFC) is a folate compound carrier protein that transports reduced folate compounds from outside into cells. This molecule also transports PEM into cells more easily than folic acid, at the same level as 5-methyltetrahydrofolate [14]. The *SLC19A1* gene has polymorphisms and was reported to be a gene predictive of the survival outcome of PEM-based chemotherapy in advanced NSCLC patients [15]. Regarding folate transport, proton-coupled folate transporter (SLC46A1/PCFT) also promotes the uptake of folates [16, 17]. The function of SLC46A1 can be optimized at an acidic pH because the flow of folates and protons into the cells depends on the proton gradient. In addition, folate receptor 1 (FOLR1/FRα) binds to oxidized folates in caveolae by bringing those folates into the cells with protons via uptake transporters in the caveolae [18].

Polyglutamate forms of folates and antifolates are catalyzed by folylpolyglutamate synthetase (FPGS) [19, 20]. A single nucleotide polymorphism of FPGS is a predicted marker of the efficacy of PEM treatment with platinum drugs in NSCLC [21]. Several other targets have also been identified, including dihydrofolate reductase (DHFR), phosphoribosylglycinamide formyltransferase (GART), ATP-binding cassette, sub-family C, member proteins 1-5 (ABCC1-5), ATP-binding cassette, sub-family C, member proteins 7 and ATP-binding cassette sub-family G member 2. [7, 22–29]. Among these target molecules, TYMS has been revealed to be responsible for PEM resistance of NSCLC [6, 8] and most predicted protein as the marker of susceptibility to pemetrexed. However, not only TYMS, any other protein has not been used as the marker in clinical setting commonly. It means the resistance mechanisms of PEM-treated NSCLC have not been found in detail, especially in the case of PEM-treated EGFR-mutated NSCLC.

In this study, we explored new drug resistance mechanisms of PEM-treated NSCLC by comparing two combinations of parental and PEM-resistant NSCLC cell lines, A549 and PC-9.

## RESULTS

### PEM sensitivity of parental and PEM-resistant NSCLC cell lines

**Figure 1:**
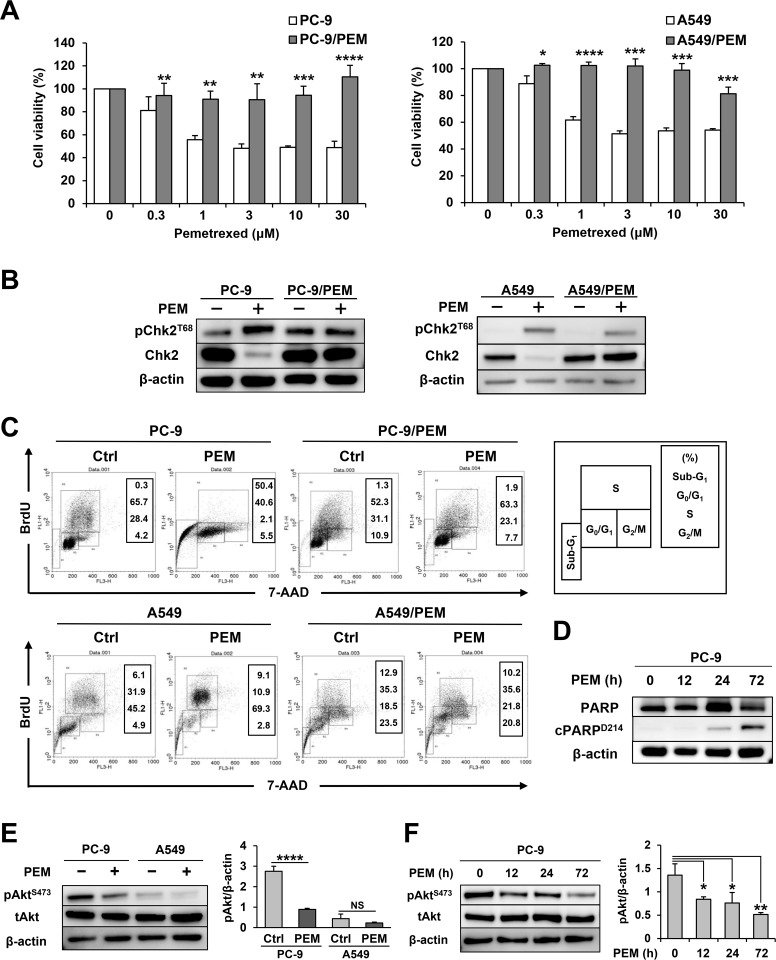
Two combinations of parental and PEM-resistant NSCLC cell lines **(A)** Parental and PEM-resistant NSCLC cell lines were treated with the indicated concentrations of PEM for 72 h (PC-9) or 96 h (A549). The cell viabilities were determined by WST-8 assay. The means ± SD are shown. N = 3, ^*^P < 0.05, ^**^P < 0.01, ^***^P < 0.001, ^****^P < 0.0001. **(B)** The total Chk2 and phosphorylated Chk2 (Thr68) protein levels in NSCLC cells treated with 3 μM PEM for 72 h (PC-9) or 96 h (A549) (+) and in untreated control cells (−). **(C)** NSCLC cell lines were cultured with 3 μM PEM or without PEM (Ctrl) for 72 h (PC-9) or 96 h (A549). Cell cycles were determined after staining with BrdU and 7-AAD using flow cytometry. **(D)** The PARP and cleaved PARP (Asp214) protein levels in parental PC-9 cells treated with 3 μM PEM for the indicated period. **(E)** The total Akt and phosphorylated Akt (Ser473) protein levels in parental NSCLC cells treated with 3 μM PEM for 96 h (+) and in untreated control cells (−). The values indicate the mean ± SD. NS, not significant, N = 3, ^****^P < 0.0001. (ANOVA, Tukey's multiple comparisons test). **(F)** The total Akt and phosphorylated Akt (Ser473) protein levels in PC-9 cells treated with 3 μM PEM for the indicated period. The values indicate the mean ± SD. N = 3, ^*^P < 0.05, ^**^P < 0.01 (ANOVA, Tukey's multiple comparisons test).

PEM-resistant NSCLC cell lines were established from PC-9 and A549 and designated as PC-9/PEM and A549/PEM, respectively. Figure 1A shows their cell viability when cultured with the indicated doses of PEM. In both cases, the PEM-resistant cell lines showed greater resistance to PEM than the parental cell lines. Thymine deficiency, which is induced by antifolate drugs, imposes constitutive DNA replication stress on cells. In order to confirm whether PEM induces the DNA damage response in these parental and resistant cell lines, we checked the phosphorylation status of Chk2^T68^ (Figure 1B). While phosphorylated Chk2 was slightly increased in PEM-treated A549/PEM cells, we confirmed that phosphorylated Chk2 increased and total Chk2 decreased in those parental cell lines alone. This finding suggested that PC-9/PEM and A549/PEM resist pemetrexed by avoiding DNA damage. We next performed a flow cytometric analysis to examine the cell cycle and apoptosis (Figure 1C). PEM showed markedly different effects on PC-9 and A549 cells. PEM drastically increased the percentage of apoptotic sub-G_1_-phase subset in PC-9 cells, whereas this change was not observed in PC-9/PEM cells. In contrast, the apoptotic sub-G_1_-phase subset of A549 cells was only slightly increased from 6.1% to 9.1% after PEM treatment. However, PEM increased the proportion of the S-phase subset of A549 cells, suggesting that the excess intracellular incorporation of BrdU occurs because of thymine deficiency. In addition, this change was not observed in A549/PEM cells, which suggests that PEM did not disturb any part of the cell cycle. To confirm the presence of apoptotic PC-9 cells, we checked the PARP cleavage as a maker of apoptosis and found it to be increased in PEM-treated PC-9 cells (Figure 1D). Given that the PI3K/Akt pathway inhibits the pro-apoptotic factors such as caspase-9, we examined the effect of PEM on the activation of Akt in PC-9 cells and A549 cells. As shown in Figure 1E, PEM treatment decreased the levels of phosphorylated Akt^S473^ in PC-9 cells. In contrast, such effects were not observed in PEM-treated A549 cells. The PEM-mediated inhibition of phosphorylated Akt started 12 h after the PEM treatment (Figure 1F). These results indicate that PEM reduces the cell viability of PC-9 cells mainly via apoptosis through inhibiting the PI3K/Akt pathway, but that this reagent can decrease the cell viability of A549 cells via folates deficiency.

### PEM forced A549 cells into senescence

**Figure 2:**
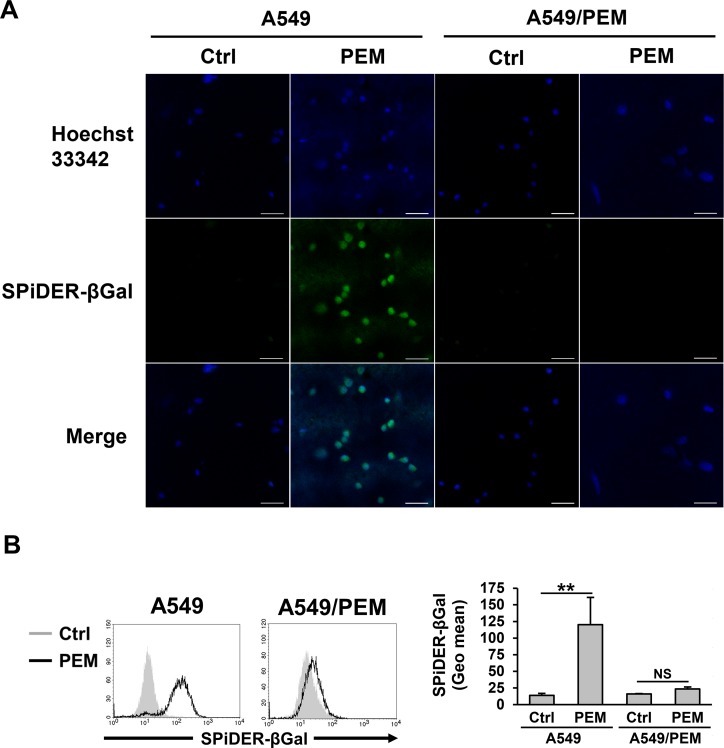
PEM-induced senescence in the parental A549 cell line **(A)** A fluorescence image of A549 and A549/PEM cells stained with SPiDER-βGal after treatment with 3 μM PEM (96 h) or untreated (Ctrl). Scale bar = 50 μm. **(B)** Flow cytometry of A549 and A549/PEM cells stained with SPiDER-βGal after treatment with 3 μM PEM (96 h) and of untreated cells (Ctrl). The bars represent the mean ± SD. N = 3; NS, not significant, ^**^P < 0.01 (ANOVA, Tukey's multiple comparisons test).

Although PEM-treated A549 cells stopped the cell cycle at the intra-S phase, we wondered why the parental A549 cells had higher viability than the parental PC-9 cells after PEM treatment (Figure 1A and 1C). This result suggests that the cell metabolism of the PEM-treated A549 cells was altered but not stopped. To confirm the reaction of A549 cells in response to PEM, we observed senescent cells, which are in a state of permanent proliferative arrest. While PEM induced senescence in the parental A549 cells, as judged by the increased numbers of senescence-associated beta-galactosidase (SA-β-Gal)-stained cells, no marked differences were observed in PEM-treated or untreated A549/PEM cells (Figure 2A). A fluorescence intensity analysis was performed using flow cytometry after PEM treatment (Figure 2B). Surprisingly, almost all of the surviving PEM-treated A549 cells were senescent whereas there was no significant difference between PEM-treated and untreated A549/PEM cells. These results show that PEM induces senescence in NSCLC cells, and that the A549/PEM cell line is resistant to PEM-induced senescence.

### The deficient expression of SLC19A1 mRNA and PEM-specific resistance

**Figure 3:**
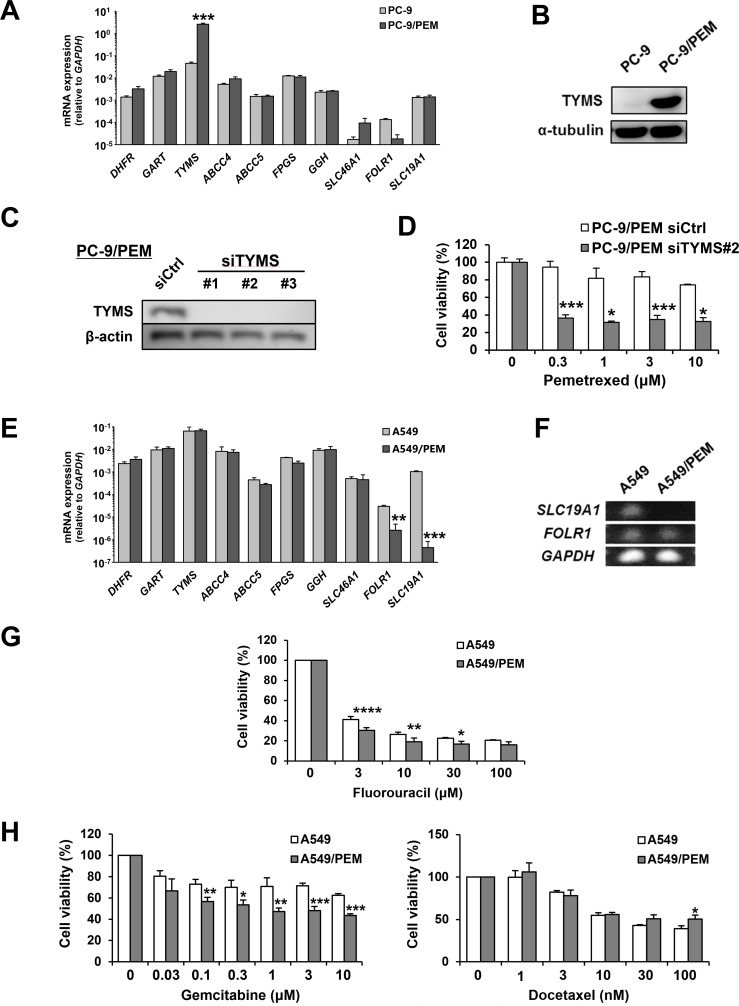
Two different variations of PEM-resistant cell lines **(A)** The mRNA expression of genes related to the mechanism of action of PEM in PC-9 and PC-9/PEM cells was assessed by an RT-qPCR. The means ± SD of the mRNA expression of each cell line are shown. N = 3, ^***^P < 0.0001. **(B)** The TYMS protein levels in PC-9 or PC-9/PEM cells were examined by immunoblotting. **(C)** PC-9/PEM cells that were transfected with negative control siRNA or TYMS siRNA. The protein levels of TYMS were examined by immunoblotting. **(D)** PC-9/PEM cells pre-transfected with control or TYMS (#2) siRNA were examined for the sensitivity to PEM. The cell viabilities were determined by WST-8 assay. The means ± SD are shown. N = 3, ^*^P < 0.05, ^***^P < 0.001. **(E)** The mRNA expression of genes related to the mechanism of action of PEM in A549 and A549/PEM cells was assessed by an RT-qPCR. The means ± SD of the mRNA expression of each cell line are shown. N = 3, ^**^P < 0.001, ^***^P < 0.0001. **(F)** An agarose-gel electrophoresis image of the *SLC19A1* and *FOLR1* PCR products of the RT-qPCR. *GAPDH* expression was used as a control. **(G)** A549 and A549/PEM cell lines were treated with fluorouracil (48 h). The cell viabilities were determined by WST-8 assay. The means ± SD are shown. N = 3, ^*^P < 0.05, ^**^P < 0.01, ^****^P < 0.0001. **(H)** A549 and A549/PEM cell lines were treated with gemcitabine (48 h) or docetaxel (48 h). The cell viabilities were measured by WST-8 assay. The means ± SD are shown. N = 3, ^*^P < 0.05, ^**^P < 0.01, ^***^P < 0.001.

We next compared the mRNA expression of several genes considered to be involved in PEM resistance between the parental and PEM-resistant cell lines. The expression of *TYMS* mRNA was significantly increased in PC-9/PEM cells compared with the parental PC-9 cells (Figure 3A). We also confirmed the presence of a strikingly increased TYMS level in PC-9/PEM cells based on an immunoblot analysis (Figure 3B). We therefore considered the dependency of the TYMS increase on the PEM resistance of PC-9/PEM cells. After confirming the siRNA-mediated knockdown of TYMS (Figure 3C), we compared the PEM sensitivity of PC-9/PEM cells that had been pre-transfected with either control or *TYMS* (#2) siRNA. As a result, the knockdown of TYMS significantly increased the PEM sensitivity of PC-9/PEM cells (Figure 3D). On the other hand, the mRNA expression of *FOLR1* and *SLC19A1* in A549/PEM cells significantly decreased in comparison to the parental A549 cells (Figure 3E). The difference in the *SLC19A1* mRNA expression was particularly remarkable. As shown in Figure 3F, we performed electrophoresis using the PCR products of RT-qPCR to compare the mRNA expression of two genes between A549 and A549/PEM cells. Surprisingly, the PCR product of *SLC19A1* was not detected at all in A549/PEM cells, whereas there was no marked difference in the PCR product of *FOLR1*. We next examined whether A549/PEM cells have resistance to fluorouracil, another antifolate drug, and other types of anti-cancer drugs, namely docetaxel and gemcitabine. Unexpectedly, A549/PEM cells had no resistance to fluorouracil (Figure 3G). Moreover, A549/PEM cells showed increased sensitivity to gemcitabine, while the sensitivity of A549/PEM cells was slightly decreased by 100 nM docetaxel (Figure 3H). These results suggest that A549/PEM cells acquired PEM-specific resistance. Given that TYMS is known to be responsible for the PEM resistance in NSCLC cells, we mainly focused on SLC19A1 in A549 and A549/PEM cells in subsequent experiments.

### SLC19A1 has a negative role in the PEM resistance of NSCLC cell lines

**Figure 4:**
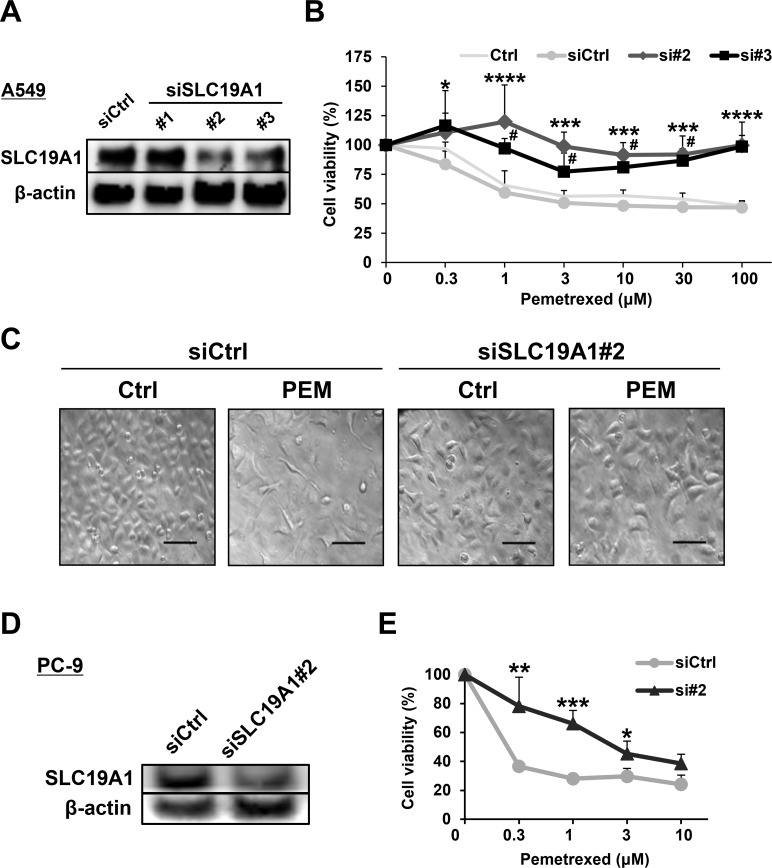
SLC19A1 negatively regulates PEM-sensitivity in NSCLC cell lines **(A)** Three types of *SLC19A1* siRNAs and negative control siRNA were transfected into A549 cells, and the SLC19A1 protein levels were examined by immunoblotting. **(B)** Negative control siRNA (siCtrl) or *SLC19A1* (#2 and #3) siRNAs was transfected into A549 cells. Control A549 cells (Ctrl) were used as a control without transfection. The cell viabilities were determined by WST-8 assay. The means ± SD are shown. Similar results were obtained in three independent experiments. N = 3, (siCtrl vs. si#2, ^*^P < 0.05, ^***^P < 0.001, ^****^P < 0.0001) (siCtrl vs. si#3, #*P* < 0.05). (ANOVA, Dunnett's multiple comparisons test). **(C)** Microscopic images (×100) of siRNA-transfected A549 cells treated with or without PEM (30 μM for 96 h). Scale bar = 100 μm. **(D)** PC-9 cells that were transfected with negative control siRNA or *SLC19A1* (#2) siRNA. The protein levels of SLC19A1 was examined by immunoblotting. **(E)** Negative control siRNA (siCtrl) or *SLC19A1* (#2) siRNA was transfected into PC-9 cells. The cell viabilities were determined by WST-8 assay. The means ± SD are shown. N = 3, ^*^P < 0.05, ^**^P < 0.01, ^***^P < 0.001.

SLC19A1 was logically important to PEM resistance because of the function of transporting PEM into cells. However, the role of SLC19A1 in PEM resistance was not well examined in NSCLC cells. Before performing experiments to examine the roles of SLC19A1 in NSCLC cells, we confirmed that *SLC19A1* siRNA (#2 and #3) transfection was able to reduce the protein expression of SLC19A1 (Figure 4A). Certainly, the siRNA-mediated knockdown of SLC19A1 significantly reduced the sensitivity of A549 cells to PEM (Figure 4B). Microscopic imaging showed that PEM significantly reduced the number of control siRNA-transfected A549 cells, while no such change was observed in *SLC19A1* siRNA-transfected A549 cells (Figure 4C). Similarly, the siRNA-mediated knockdown of SLC19A1 caused PC-9 cells to become PEM-resistant (Figure 4D and 4E). These results indicate that SLC19A1 plays a negative role in the PEM resistance of NSCLC cells.

### PC-9/PEM cells collaterally acquired EGFR-independent Akt activation with PEM resistance

**Figure 5:**
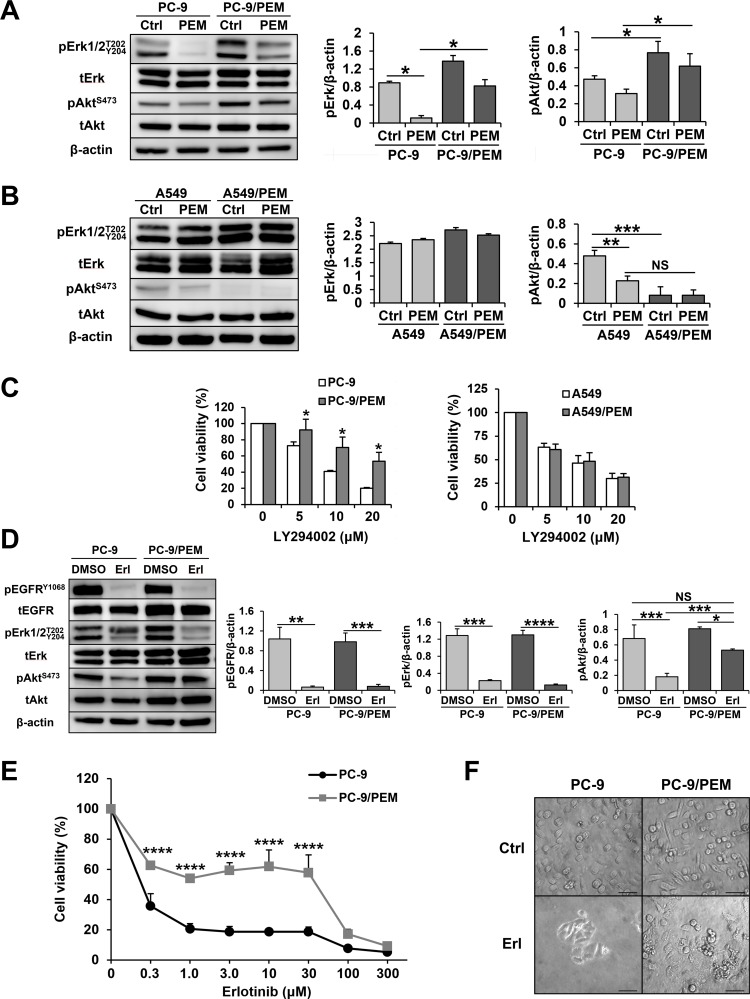
PEM treatment bestows EGFR-independent PI3K-Akt signaling activation on PEM-resistant PC-9 cells **(A)** Cell lysates of PC-9 or PC-9/PEM were used in immunoblotting after treatment with 3 μM PEM (72 h). The quantification of pErk and pAkt in PC-9 or PC-9/PEM cells by immunoblotting. N = 3, ^*^P < 0.05. (ANOVA, Tukey's multiple comparisons test). **(B)** Cell lysates of A549 or A549/PEM were used for immunoblotting after treatment with 3 μM PEM (96 h). The quantification of pErk and pAkt in A549 or A549/PEM cells by immunoblotting. N = 3; NS, not significant, ^**^P < 0.01, ^***^P < 0.001. (ANOVA, Tukey's multiple comparisons test). **(C)** Two combinations of parental and PEM-resistant NSCLC cell lines were treated with the indicated concentrations of a PI3K inhibitor LY294002. The cell viabilities were determined by WST-8 assay. The means ± SD are shown. N = 4, ^*^P < 0.05 **(D)** Cell lysates of PC-9 or PC-9/PEM were used for immunoblotting after treatment with 1 μM erlotinib or DMSO (96 h). The quantification of pEGFR, pErk and pAkt in PC-9 or PC-9/PEM cells by immunoblotting. N = 3; NS, not significant, ^*^P < 0.05, ^**^P < 0.01, ^***^P < 0.001, ^****^P < 0.0001 (ANOVA, Tukey's multiple comparisons test). **(E)** The cell viabilities were determined by WST-8 assay. Similar results were obtained in three independent experiments. The means ± SD of cell viabilities are shown. N = 4, ^****^P < 0.0001. **(F)** Microscopic images (×200) of PC-9 and PC-9PEM cells treated with or without 30 μM erlotinib for 96 h. Scale bar = 50 μm.

EGFR mutations provide an activating signal to the PI3K/Akt pathway, and PC-9 cells carry an EGFR mutation (p.E746_A750del). In addition, PEM inhibits the PI3K/Akt pathway, leading to apoptosis in PC-9 cells (Figure 1E and 1F). Figure 5A shows that PEM clearly decreased the phosphorylation levels of Erk and Akt in parental PC-9 cells. Surprisingly, in addition to the increase in TYMS, the PC-9/PEM cells also showed greater Akt activation than PC-9 cells. Furthermore, PEM-treated PC-9/PEM cells have higher levels of phosphorylated Akt than PEM-untreated PC-9. In contrast, A549/PEM cells showed less Akt activation than A549 cells that carried a KRAS mutation (Figure 5B). Thus, we next examined the dependency of the PI3K/Akt signaling in PC-9 and PC-9/PEM cells using the PI3K inhibitor LY294002. We found that PC-9/PEM cells were more resistant to the LY294002 than PC-9 cells (Figure 5C), whereas no difference was observed in A549 and A549/PEM cells. These results suggest that PC-9/PEM cells were less susceptible to PI3K/Akt-pathway inhibitors than PC-9 cells. Importantly, PC-9 has the most common mutation of EGFR, namely an exon 19 deletion. Therefore, to evaluate the efficacy of EGFR-TKI, we treated PC-9 and PC-9/PEM cells with an EGFR-TKI, erlotinib. As shown in Figure 5D, erlotinib drastically inhibited the phosphorylation of EGFR^Y1068^ and Erk^T202/Y204^ in both PC-9 and PC-9/PEM cells. Whereas erlotinib sufficiently inhibited the phosphorylation in PC-9 cells, its inhibition of the phosphorylation of Akt was insufficient in PC-9/PEM cells. This reveals that the phosphorylation of Akt in the PC-9/PEM cell line is augmented in comparison to the PC-9 cell line, regardless of the EGFR phosphorylation status. Supporting this notion was the fact that PC-9/PEM cells had greater erlotinib resistance than parental PC-9 cells (Figure 5E and 5F).

### Xenograft models of PEM-resistant tumors in BALB/c nude mice

**Figure 6:**
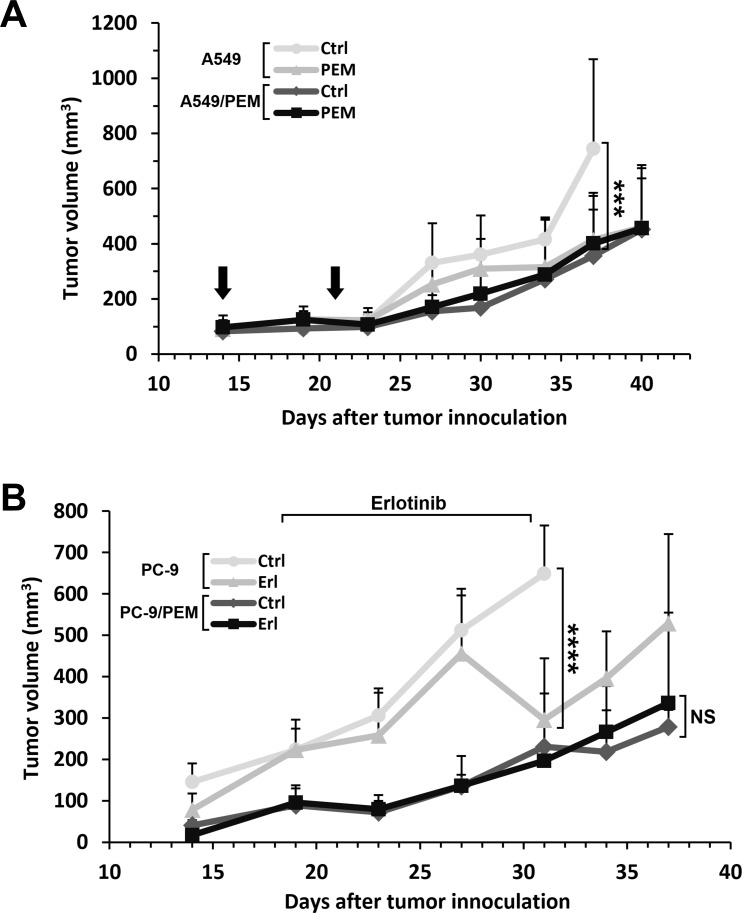
The drug responses of PEM-resistant human NSCLC tumors in nude mice treated with PEM or erlotinib **(A)** The time course of the tumor volume. A549 or A549/PEM cells were subcutaneously inoculated into the right flank of nude mice on day 0. PEM treatment (200 mg/kg) or vehicle control (Ctrl) were administered by intraperitoneal injection on days 14 and 23. The arrows indicate the days of PEM injection. The values indicate the mean tumor volume ± SD. N = 5, ^***^P < 0.001 (ANOVA, Tukey's multiple comparisons test). **(B)** The time course of the tumor volume. PC-9 or PC-9/PEM cells were subcutaneously inoculated into the right flank of nude mice on day 0. Erlotinib (Erl) (50 mg/kg) or vehicle control (Ctrl) were administered by oral gavage once daily, 6 days a week. The values indicate the mean tumor volume ± SD are shown. N = 5; NS, not significant; ^****^P < 0.0001 (ANOVA, Tukey's multiple comparisons test).

We also evaluated the drug resistance of PEM- and erlotinib-treatment in a xenograft mouse model. A549 or A549/PEM cells were subcutaneously inoculated into the right flanks with 1.5×10^6^ cells. On day 37, during the administration of pemetrexed, significant differences were observed in the A549 tumors of the control and the pemetrexed-treated groups. Although the absolute sizes were smaller than the parental A549 tumors, the sizes of the A549/PEM tumors in the control and PEM-treated group did not differ to a statistically significant extent at any point in the experimental period (Figure 6A). PC-9 and PC-9/PEM cells were used to investigate the effects of erlotinib treatment. At 2 weeks after inoculation, erlotinib was orally administered 6 days a week for 2 weeks (50 mg/kg bodyweight). Although significant differences were observed in the PC-9 tumors of the control and erlotinib-treated groups during the administration period, no significant differences were found in the PC-9/PEM tumors (Figure 6B).

## DISCUSSION

We revealed for the first time that SLC19A1 negatively regulates PEM resistance in NSCLC cells (Figure 4B and 4E). Additionally, we found that PEM resistance is associated with EGFR-TKI resistance in EGFR-mutated NSCLC cells through EGFR-independent Akt activation (Figure 5D and 5E).

In this study, we explored new drug-resistance mechanisms of PEM-treated NSCLC by comparing two combinations of parental and PEM-resistant NSCLC cell lines. We found that the PC-9/PEM cells had increased expression of *TYMS* and that A549/PEM cells had decreased expression of *SLC19A1* compared with the respective parental cell lines. Given that TYMS was already known to be responsible for PEM resistance of NSCLC [8], we focused on SLC19A1. A previous study reported that a murine leukemia cell line acquired PEM resistance by decreasing the expression of SLC19A1 and *FPGS* without an increase in the *TYMS* expression [30]. A single nucleotide polymorphism (SNP) of the *SLC19A1* gene was reported to induce a difference in the transport and toxicity of methotrexate, an antifolate drug inhibiting DHFR, in patients with hematological malignancies [31]. Transcriptional silencing, inactivating mutations and allele loss of *SLC19A1* decreased the MTX influx in human leukemia cells [32]. In addition, the genomic deletion of *SLC19A1* increases the IC_50_ of PEM in human cervical cancer HeLa cells [33]. In particular, another SNP of *SLC19A1* significantly influenced the overall survival in patients with advanced NSCLC [15]. Taken together, these findings suggest that SLC19A1 plays crucial roles in PEM resistance of several cancer types. However, to our knowledge, no report has shown that SLC19A1 negatively regulates PEM resistance of human NSCLC.

PEM is an antifolate drug, and there are several mechanisms by which cells take up folate [34]. How can NSCLC cells exert resistance to PEM by reducing their SLC19A1 expression? SLC19A1 not only helps cells take up PEM but also delivers folates into the cytoplasm [35]. Therefore, the decreased expression of SLC19A1 would consequently reduce the amount of folate, which is needed for cell proliferation [35]. However, there are compensatory systems to take up folate. Without SLC19A1, cells can take up folate via other transport molecules, including FOLR1 and SLC46A1. FOLR1 captures folates and transports them into cells via receptor-mediated endocytosis. Acidic conditions in endosomes help SLC46A1 function. SLC46A1 and FOLR1 are thought to work in cooperation. Therefore, the decreased expression of SLC19A1 is actually convenient for the survival of cancer cells, decreasing the uptake of PEM while maintaining the uptake of folate. Importantly, this same mechanism was also reported in another NSCLC PC-9 cell line, suggesting that this phenomenon may be generalized.

The cytotoxic effect of pemetrexed is still unresolved, even though pemetrexed is an antifolate agent widely used to treat NSCLC and malignant pleural mesothelioma. In fact, A549 cells behaved differently from PC-9 cells after PEM treatment and this difference was unexpected. We performed a cell cycle analysis and SA-β-Gal staining, which revealed that thymine deficiency and cell senescence were induced in PEM-treated A549 cells. Cellular senescence is an intrinsic anticancer mechanism that prevents aberrant cell cycle progression. Cellular senescence is widely induced by radiation therapy, reactive oxygen species and several anti-cancer drugs [36, 37]. In addition, senescent cells remain, and activate the cellular metabolism to secrete proinflammatory molecules (known as the senescence-associated secretory phenotype [SASP]). The results of the cell viability assay and SA-β-Gal staining suggest that PEM-treated A549 cells have an active metabolism that produces SASP. PEM-induced senescence may be one of the reasons why the anticancer effects of PEM are maintained for long periods during continuous chemotherapy. For instance, if every drug-susceptible cell was killed and removed after treatment, the remaining drug-resistant cells could grow, making use of the empty niche. On the other hand, drug-induced senescent cells typically become flattened and enlarged in comparison to untreated cells and thus do not create room for growing cells. Elucidating this mechanism would shed light on the role of senescence in the evolution of drug resistance.

On the other hand, PEM decreased the phosphorylation of Akt^S473^ and increased apoptotic cells in PC-9 cells. PC-9 cells carry the EGFR exon 19 deletion, leading to the activation of the PI3K/Akt pathway for tumorigenic proliferation [38, 39] and this activation plays a crucial role in preventing apoptosis in cancerous cells [40]. PEM decreased the phosphorylation of Akt in PC-9 cells, and it was also reported that PEM can inhibit this pathway [29, 41, 42]. Interestingly, in comparison to PC-9 cells, the phosphorylation of Akt was greatly enhanced in PC-9/PEM cells due to their PEM resistance. In addition, PC-9/PEM cells were more resistant than parental PC-9 cells to the EGFR-TKI erlotinib inhibiting the PI3K/Akt pathway [43]. Collectively, PEM resistance is positively associated with erlotinib resistance in the case of EGFR mutation-carrying NSCLC cells. In support of this, PC-9/PEM cells were more resistant to the PI3K/Akt inhibitor LY294002 than parental PC-9 cells. As we are unsure about the association between PEM resistance and EGFR-TKI resistance in the clinical setting at present, this should be considered carefully when treating patients with NSCLC carrying an EGFR mutation with PEM and/or EGFR-TKI. In the clinical setting, Xu et al. reported that the administration of EGFR-TKIs as a first-line therapy was more beneficial than the administration of EGFR-TKIs as a second-line after chemotherapy in patients with EGFR-sensitive mutations [44]. Moreover, the increased phosphorylation of Akt was reported to be a convergent feature of EGFR-TKI resistance and a novel biomarker that predicted a decreased initial EGFR-TKI response [45]. Although the precise mechanism through which this occurs in the clinical setting remains unknown, the emergence of EGFR-TKI resistance similar to the resistance of PC-9/PEM could be one reason.

In conclusion, we identified new drug resistance mechanisms of PEM-treated NSCLC cells. Our findings showed that SLC19A1 negatively regulates PEM resistance in NSCLC cells. In addition, EGFR-TKI inhibitor resistance was observed to occur with PEM resistance in EGFR mutation-carrying NSCLC cells via an up-regulated Akt activation. Given these findings, we plan to examine the SLC19A1 expression and phosphorylation of Akt in tumor tissues from NSCLC patients before and after PEM treatment to evaluate the effects that such factors have on clinical efficacy.

## MATERIALS AND METHODS

### Cell lines and reagents

Two human lung adenocarcinoma cell lines were used: PC-9, a differentiated human lung adenocarcinoma cell line; and A549, a human lung adenocarcinoma cell line derived from epithelial cells. PC-9 has an EGFR exon 19 deletion mutation (p.E746_A750del), and A549 has a KRAS mutation (p.G12S). These cell lines were maintained in RPMI-1640 medium (Wako, Osaka, Japan), which was supplemented with 10% (v/v) fetal bovine serum (FBS) and 50 μg/mL gentamicin in a humidified CO_2_ incubator (37 °C, 5% CO_2_). Both cell lines were tested and authenticated through genetic testing by using PowerPlex 16 STR System by the National Institute of Biomedical Innovation, Osaka, Japan in July 2016. PEM was obtained from Eli Lilly Japan K.K., Hyogo, Japan, and diluted with phosphate-buffered saline (PBS). LY294002 and erlotinib were purchased from SA Bioscience (Frederick, MD, USA) and Chugai Pharmaceutical (Tokyo, Japan), respectively, and diluted with dimethyl sulfoxide (DMSO).

### Establishment of PEM-resistant NSCLC cell lines

To establish PEM-resistant NSCLC cell lines, cancer cells were continuously cultured in the presence of PEM. When culturing the cells, we used several culture dishes. Each time, we chose the dish with the greatest number of growing cells to passage. We aimed to increase the PEM concentration to at least 3 μM (this concentration was based on the area under the blood concentration-time curve from a phase I study [46]) to induce sufficient resistance in every culture condition used in this study. To establish the cell lines, the cell lines were passaged approximately 100 times. The final PEM concentrations for the PEM-resistant PC-9 and A549 cell lines were ≥3 μM (3.0 and 4.0 μM, respectively). These cell lines were designated PC-9/PEM and A549/PEM.

### Cell viability assay

To measure the cell viability, cancer cells were cultured with the indicated doses of drugs in 96-well tissues culture plates. The cell viability was determined by WST-8 assay using a Cell Counting Kit-8 (Dojindo Laboratories, Kumamoto, Japan). The absorbances at 450 nm were measured using a 96-well microplate reader. The absorbance of each well with drugs was divided by the standard without any drugs to give the relative cell viability (%).

### Reverse transcription quantitative polymerase chain reaction (RT-qPCR)

Cells at around 80% confluence were washed with PBS. The total RNAs of these cells were extracted using an RNeasy Mini Kit (Qiagen, Tokyo, Japan), in accordance with the manufacturer's instructions. cDNAs were generated from the RNAs via reverse transcription using ReverTra Ace qPCR RT Master Mix with gDNA remover (TOYOBO, Osaka, Japan), in accordance with the manufacturer's instructions. qPCR was performed using KOD SYBR qPCR Mix (TOYOBO). The primers used for qPCR were as follows:

*DHFR* primers 5′- CCATACTGCTGAGATACAGG GAAAT -3′ and 5′- ACACAGGACAGGGAGCTGACA -3′;

*GART* primers 5′- CAATGGCAGCCCGAGTACT TA -3′ and

5′- GACATGATGAGACTGTGCAAGTTTC -3′;

*TYMS* primers 5′- CACACTTTGGGAGATGCACA TATT -3′ and

5′- TTCGAAGAATCCTGAGCTTTGG -3′;

*ABCC4* primers 5′- TCTGGACCATCCGGGCATAC -3′ and

5′- TGGTGGTGGGCGTTTCTGAT -3′;

*ABCC5* primers 5′- CCTGCAGTACAGCTTGTTG TTAGTG -3′ and

5′- GACACCGGTTCGGTAATTCAAT -3′;

*SLC46A1* primers 5′- CTGGACCCTCTACATG AACG -3′ and 5′- GGTAGAGTGAGTTGAAGATG -3′;

*FPGS* primers 5′- CTATGCCGTCTTCTGCCCTAAC -3′ and 5′- ACCTGGTCCAGTGTCACTGTGA -3′;

*GGH* primers 5′- GCGAGCCTCGAGCTGTCTA -3′ and 5′- AATATTCCGATGATGGGCTTCTT -3′;

*FOLR1* primers 5′- AGGACAAGTTGCATGA GCAGTG -3′ and 5′- TCCTGGCTGGTGTTGGTAG -3′;

*SLC19A1* primers 5′- CATCGCCACCTTTCAGATT -3′ and 5′- TGGCAAAGAACGTGTTGAC -3′;

*GAPDH* primers 5′- GCACCGTCAAGGCTGAG AAC -3′ and 5′- TGGTGAAGACGCCAGTGGA -3′.

The *GAPDH* expression was used as a standard to normalize the relative expressions. PCR was performed for 45 cycles at 90 °C for 15 s and at 60 °C for 1 min.

### Immunoblot protein analysis

Harvested cells were washed by PBS and lysed using M-PER Mammalian Protein Extraction Reagent (Thermo Fisher Scientific, Waltham, MA, USA) with 1% Protease Inhibitor Cocktail (Thermo Fisher Scientific) and 1% Phosphatase Inhibitor Cocktail (Nacalai Tesque, Inc., Kyoto, Japan). The lysates were centrifuged at 14,000 *g* for 15 min. The supernatants were collected as protein samples. The protein concentrations in the samples were measured with the Coomassie Plus Bradford Protein Assay Kit (Thermo Fisher Scientific). These samples and Bolt LDS Sample Buffer (Thermo Fisher Scientific) and Bolt Sample Reducing Agent were mixed and heated at 70 °C for 10 min to denature proteins. Denatured proteins which were the same volumes and the same concentrations as that of the total proteins were loaded onto a Bolt 4-12% Bis-Tris Plus Gel (Thermo Fisher Scientific). The proteins were then transferred to the gel using a Bolt Mini Gel Tank (Thermo Fisher Scientific), and each separated protein on the gel was transferred to an iBlot Transfer Stacks PVDF mini (Thermo Fisher Scientific) on an iBlot Dry Blotting System (Thermo Fisher Scientific). The following antibodies were used as the primary antibodies: anti-SLC19A1 (GeneTex, Irvine, CA, USA), anti-TYMS (M3614; Agilent Technologies Japan, Tokyo, Japan), anti-tChk2, anti-pChk2^T68^, anti-PARP, anti-cPARP^D214^, anti-tEGFR, anti-pEGFR^Y1068^, anti-pAkt^S473^, anti-tErk1/2, anti-pErk1/2^T202/Y204^ (Cell Signaling Technology, Danvers, MA, USA), anti-tAkt, anti-α-tubulin (Santa Cruz Biotechnology, Inc. CA, USA), anti-β-actin (BioLegend, San Diego, CA, USA) antibodies. Secondary Antibody Solution Alk-Phos Conjugated (Thermo Fisher Scientific) was used as the alkaline phosphatase conjugated secondary antibody. Novex AP Chemiluminescent Substrate (Thermo Fisher Scientific) was used as the substrate for alkaline phosphatase. Anti-Rabbit IgG, HRP-Linked Whole Ab Donkey (GE Healthcare UK Ltd, Buckinghamshire, England) was used as the horseradish peroxidase conjugated secondary antibody. ECL Select Western Blotting Detection Reagent (GE Healthcare UK Ltd) was used as the substrate for horseradish peroxidase. The density was quantified as the ratio of each intensity band as quantified by the Image J software program (version 1.51j8, NIH).

### Cell cycle analysis

To examine the cell cycle and proliferation of cancer cells, a BrdU Flow Kit (Becton, Dickinson and Company, Franklin Lakes, NJ, USA) was used in accordance with the manufacturer's instruction. Cell lines were incubated with BrdU (10 μM) in RPMI-1640 media for 150 min before staining with FITC-conjugated anti-BrdU antibody. Flow cytometry was performed using a BD FACSCalibur (Becton, Dickinson and Company).

### Senescence-associated beta-galactosidase staining

SPiDER-βGal was purchased from Dojindo Laboratories. Cells were cultured in 8-well glass chamber slide (Becton, Dickinson and Company). Culturing cells were stained with Hoechst 33342 (Dojindo Laboratories) before SA-β-Gal staining. For SA-β-Gal staining, cells were fixed with 4% (w/v) paraformaldehyde for 5 min and stained with SPiDER-βGal working solution at 37°C for 30 min. All images were obtained with a laser scanning confocal fluorescence microscope FV1000D (Olympus, Tokyo, Japan). Flow cytometry analysis was performed using a BD FACSCalibur (Becton, Dickinson and Company). Geometric means were quantified by using CellQuest Pro software (Becton, Dickinson and Company).

### Small interfering RNA transfection

Small interfering RNA (siRNA) experiments were performed using Silencer Select Negative Control siRNA (cat#4390844) for a negative control. Pre-Designed Silencer Select siRNAs were used against each gene. Against *SLC19A1*, si#1 (ID#s13084), si#2 (ID#s13085) and si#3 (ID#s13086) were used. Against *TYMS,* siTYMS#1 (ID#s14538), siTYMS#2 (ID#14539) and siTYMS#3 (ID#14540) were used. All siRNAs were obtained from Thermo Fisher Scientific. One volume percent of Lipofectamine RNAiMax Reagent (Thermo Fisher Scientific) and siRNA solutions were dissolved in Opti-MEM Media (Thermo Fisher Scientific) in the wells of 6-well plates and then incubated for 30 min. A total of 5×10^4^ cells of A549 or 1.5×10^5^ cells of PC-9 were seeded per well in 6-well plates at a final concentration of 6 nM siRNA. Cells were collected at 48 h after transfection and used for other experiments.

### Animal experiment

The protocols of all animal experiments were approved by the Committee for Animal Experimentation of Shimane University, and met the ethical standards required by law and the guidelines on animal experiments in Japan (IZ29-63). BALB/c nu/nu mice were purchased from Clea Japan. Male mice of >8 weeks of age were used in all experiments. The mice were monitored for symptoms of illness by investigating changes in weight, activity, and skin texture at least twice a week. Every cell line was subcutaneously inoculated into the right flank with 1.5×10^6^ cells.

### Statistical analysis

Statistical analyses were conducted using the IBM SPSS Statistics software program, version 20 (IBM, Armonk, NY, USA). The distribution of all data was distinguished using the Shapiro-Wilk test. For comparing the differences between two groups, Student's unpaired two-tailed *t*-test was used for normally distributed data (*P* < 0.05), while the Mann-Whitney test was used for non-normally distributed data. *P* < 0.05 and *P* < 0.01 were considered significant in the experiments of cell viability assay and in the RT-qPCR analysis, respectively. An ANOVA and a post-hoc analysis were used to compare the differences among more than two groups.

## Abbreviations

(NSCLC): non-small cell lung cancer
(EGFR): epidermal growth factor receptor
(TKI): tyrosine kinase inhibitor
(PEM): pemetrexed
(TYMS): thymidylate synthase
(folate transporter): solute carrier family 19
(SLC19A1/RFC): member 1
(SLC46A1/PCFT): proton-coupled folate transporter
(FOLR1/FRα): folate receptor 1
(FPGS): folylpolyglutamate synthetase
(DHFR): dihydrofolate reductase
(GART): phosphoribosylglycinamide formyltransferase
(ABCC1-5): ATP-binding cassette, sub-family C, member proteins 1-5
(SA-β-Gal): senescence-associated beta-galactosidase
(SASP): senescence-associated secretory phenotype
